# Logogenic Primary Progressive Aphasia or Alzheimer Disease: Contribution of Acoustic Markers in Early Differential Diagnosis

**DOI:** 10.3390/life12070933

**Published:** 2022-06-22

**Authors:** Eloïse Da Cunha, Alexandra Plonka, Seçkin Arslan, Aurélie Mouton, Tess Meyer, Philippe Robert, Fanny Meunier, Valeria Manera, Auriane Gros

**Affiliations:** 1 Speech Therapy Department of Nice, Faculty of medicine, Université Côte d’Azur, 06000 Nice, France; alexandra.pl@gmail.com (A.P.); mouton.a2@chu-nice.fr (A.M.); tess_meyer@live.fr (T.M.); philippe.robert@univ-cotedazur.fr (P.R.); auriane.gros@univ-cotedazur.fr (A.G.); 2 Laboratoire CoBTeK (Cognition Behaviour Technology), Université Côte d’Azur, 06000 Nice, France; valeria.manera@univ-cotedazur.fr; 3 Institut NeuroMod, Université Côte d’Azur, 06902 Sophia-Antipolis, France; 4 Service Clinique Gériatrique du Cerveau et du Mouvement, CMRR, Centre Hospitalier Universitaire, 06000 Nice, France; 5 BCL, CNRS UMR7320, Campus Saint Jean d’Angely—SJA3/MSHS-SE, Université Côte d’Azur, 06300 Nice, France; seckin.arslan@univ-cotedazur.fr (S.A.); fanny.meunier@unice.fr (F.M.)

**Keywords:** primary progressive aphasia, logopenic variant, Alzheimer’s disease, diagnosis, acoustic markers, prosody, early markers

## Abstract

The logopenic variant of Primary Progressive Aphasia (lvPPA), a syndromic disorder centered on language impairment, often presents variable underlying neurodegenerative pathologies such as Alzheimer Disease (AD). Actual language assessment tests and lumbar puncture, focused on AD diagnosis, cannot precisely distinguish the symptoms, or predict their progression at onset time. We analyzed acoustic markers, aiming to discriminate lvPPA and AD as well as the influence of AD biomarkers on acoustic profiles at the beginning of the disease. We recruited people with AD (n = 8) and with lvPPA (n = 8), with cerebrospinal fluid biomarker profiles determined by lumbar puncture. The participants performed a sentence repetition task that allows assessing potential lvPPA phonological loop deficits. We found that temporal and prosodic markers significantly differentiate the lvPPA and AD group at an early stage of the disease. Biomarker and acoustic profile comparisons discriminated the two lvPPA subgroups according to their biomarkers. For lvPPA with AD biomarkers, acoustic profile equivalent to an atypical AD form with a specific alteration of the phonological loop is shown. However, lvPPA without AD biomarkers has an acoustic profile approximating the one for DLFT. Therefore, these results allow us to classify lvPPA differentially from AD based on acoustic markers from a sentence repetition task. Furthermore, our results suggest that acoustic analysis would constitute a clinically efficient alternative to refused lumbar punctures. It offers the possibility to facilitate early, specific, and accessible neurodegenerative diagnosis and may ease early care with speech therapy, preventing the progression of symptoms.

## 1. Introduction

To preserve the autonomy of the geriatric population with neurodegenerative decline, speech therapy is the most proven and useful intervention which maintains functional communication [1]. Nonetheless, speech therapy is efficient if care is initialized at the onset time of the pathology and if it is based on a specific diagnosis. Primary Progressive Aphasia (PPA) syndrome presents a debated diagnostic classification and its links with Alzheimer’s disease (AD) are still debated today [2,3]. Therefore, the imprecisions regarding the distinction between PPA and AD obstruct their differential and specific diagnosis. In this context, care is delayed and the prognosis of maintained autonomy is reduced [4]. Consequently, early specific diagnosis of neurodegenerative pathologies is crucial for adapting non-pharmacological interventions.

PPA is a neurodegenerative syndrome characterized by an isolated language impairment [5,6,7]. The consensual diagnosis criteria is the presence of aphasia as the primary cause of deficits in daily living activities, at the onset and in the initial phases of the disease [5]. Moreover, PPA affects more men than women, contrary to other neurodegenerative pathologies such as AD [4,8]. These clinical and demographic particularities differentiate PPA from other neuropathological diseases despite its heterogeneity [6,7,9]. The consensus classification of PPA distinguishes three subtypes under their clinical and neuropathological particularities: the non-fluent/agrammatic variant (nfvPPA), the semantic variant (svPPA) and the logopenic variant (lvPPA). Some non-classified groups persist, such as mixed forms [10,11,12,13]. In the evolution of PPA, language impairment remains the most characteristic symptom [6,11]. As it evolves, deficits can extend to other cognitive capacities such as psychiatric, neurologic and/or praxis capacities [13,14]. Most PPA cases progress to global neurodegenerative pathologies [9]. The incidence of the underlying pathology type varies according to the PPA subtype [9]. According to the literature, svPPA is the most homogeneous subtype, with on average 85.7% of frontotemporal lobar degeneration (FTLD) and 14.3% of AD [2,15]. Similarly, nfvPPA shows 86.1% FTLD, 5.6% AD, and 8.3% other pathologies such as Lewi-body dementia [2,16,17]. Finally, the major underlying pathology for lvPPA is AD with on average 55.6% of lvPPA cases, but it is, at this moment unpredictable: 35.5% of lvPPA degenerates as FTLD variants and 8.9% as other pathologies such as Lewi-body dementia [2].

Even if the underlying pathology can vary, clinical markers in lvPPA are most often associated with underlying early-onset AD [17,18]. In both pathologies, anomia and selective short term memory deficits are observed [11,18,19,20]. Speech output is affected by long word-finding pauses potentially caused by lexical retrieval impairment [9,11,19,20]. In lvPPA, language impairments are often blended with episodic memory deficits, one of the first AD symptoms observed [17,18,19]. At an advanced stage of lvPPA, the deficit radiates to other general cognitive abilities which are often impaired in AD, such as visuospatial abilities, memory, attention, and apathy [6]. lvPPA decline is atypical from other PPA subtypes in its fast progression of language disorders and the appearance of secondary cognitive impairments [18]. Furthermore, similarities in cortical atrophy patterns are oftentimes observed in AD and lvPPA: atrophy in the posterior superior temporal and middle temporal gyri and inferior parietal lobe, associated with the phonological loop and naming functions, and posterior parietal or Perisylvian hypometabolism [9,21,22]. Neuro-anatomic alterations in lvPPA have increasing similarities with the amnestic AD neuro-anatomic alterations described in the literature [21,23].

Consequently, the clinical and neurological evolution of lvPPA is close to an atypical AD phenotype, which highly complicates differential diagnosis [22,23,24]. Clinical and language assessments based on lvPPA clinical criteria are not sensitive enough to distinguish these pathologies [10,25]. Some differences are noticed in clinical tests, such as non-verbal episodic memory or repetition tasks, but the difference is not sufficient to obtain a significant classification [10,26]. Currently, we succeed in precise early diagnosis of PPA subtypes and AD thanks to the association of neuro-anatomic analyses with Cerebrospinal Fluid (CSF) biomarkers [27,28,29,30]. Indeed, a decrease of β-Amyloid markers and a high tau concentration confirms AD pathology [30]. Nonetheless, 60 to 90% of lvPPA patients present β-Amyloid AD markers in their CSF [10,31]. CSF biomarkers, then, cannot be a sufficient diagnostic measure to differentiate AD and lvPPA at the onset time of cognitive decline. Nonetheless, precision on PPA classification and, specifically, the sub-classification of lvPPA and its links with an underlying AD is achievable: an amyloid-negative subtype of lvPPA (−lvPPA) and an amyloid-positive sub-type (+lvPPA) have been described according to CSF biomarkers concentration [32]. The sub-type +lvPPA would present an underlying onset of AD behind the lvPPA syndrome [10,25,33,34]. Neuroimaging studies support this hypothesis, showing AD neuroparticularities in +lvPPA: a more prominent hypometabolism of the left temporoparietal region than in −lvPPA [10,25,34]. Neurologic particularities in −lvPPA consist of lower hypometabolism in the left temporo-parietal region. Hypometabolism is also extended to the anterior temporal and basal frontal regions in the −lvPPA subtype [32]. This would explain the heterogeneity of the lvPPA group and its links with AD.

Lumbar puncture is the most effective intervention for extracting CSF and obtaining significant biomarker concentrations [29,33]. Despite its great diagnostic sensitivity and specificity, the lumbar puncture is an invasive procedure with possible side effects [35]. It is often refused by patients during diagnosis searches because of its potential side effects [36]. An inequality of accessibility depending on place of residence and the expensive cost have been raised as obstructions to early diagnosis [29,35]. Nonetheless, CSF biomarkers, associated with clinical and neuroimage analyses, remain the most sensitive and specific diagnostic tool to discriminate AD at initial phases from other neurodegenerative pathologies [37].

Thus, the early differentiation of heterogeneous forms of lvPPA from AD has not yet been achieved. Nowadays, the presence of language impairment is one of the principal clinical markers for the diagnosis of PPA subtypes. Particularly, sentence repetition tasks are the most relevant clinical tools for discriminating lvPPA from other PPA subtypes, with different performances compared to people with AD [25,26]. Language assessment scores are not always sufficient for a sensitive and specific diagnosis [26]. Nonetheless, new technologies have led to the development of improved, more sensitive, specific and non-invasive tests [38,39]. Automated data analysis permits precise language analysis during assessment, using acoustic and vocal features [40]. Certain vocal and acoustic alterations cannot be perceived by human natural hearing but can be detected by automatic analyses, which have the potential to reveal discriminant features making the diagnosis more accurate and efficient [41,42]. Automated acoustic and vocal analysis has been described as a non-invasive representative marker of AD or PPA subtypes, distinguishing them from other neurodegenerative diseases at onset-time [41,43]. Specific temporal and acoustic features have been described for both lvPPA and AD voices [40,44]. Sentence repetition is also the most relevant acoustic feature for voice analysis and the most sensitive task focusing on language-specific selective impairment in the phonological loop in lvPPA [45].

Therefore, the aim of the present study is to identify vocal and acoustic markers discriminating lvPPA and AD and to analyze their impact in diagnosis as well as the influence of AD biomarkers on vocal and acoustic characteristics in a sentence-repetition task at an early stage of neurodegenerative decline.

## 2. Materials and Methods

### 2.1. Ethics

This study was approved by CPP Ile de France X (N° IDRCB: 2019-A00342-55 accepted on 11 September 2019). At the time of diagnosis, patients and relatives were informed of their inclusion in this study and could decline their participation or withdraw consent. Data was anonymized before the analyses.

### 2.2. Population

This prospective monocentric study recruited patients at Claude Pompidou Institute, the Research, Resources and Memory Center (CMRR) in Nice. Patients were recruited from memory consultations from October 2020 to March 2022. Patients had to correspond to the following inclusion criteria: being of age 40 or over, having been diagnosed of lvPPA or AD according to Gorno-Tempini classification and DSM-5TM criteria [11,46], speaking, reading and writing French fluently, having consulted the CMRR because of cognitive behavioral and/or motor difficulties, having French insurance coverage, having no objections to inclusion after reading the information note. The exclusion criteria for this study were: the existence of protective measures such as guardianship or curatorship, medication prescription with side effects on the central nervous system or interfering with the test execution or results, cerebrovascular disease history, psychiatric disorder history according to DSM-4TR criteria, any neurological pathology except lvPPA and AD, traumatic brain injury, untreated metabolic trouble, uncorrected hearing and vision problems and/or refusal to undergo paramedical examination such as lumbar puncture, MRI (Magnetic Resonance Imagery) and/or PET-SCAN (fluorodeoxyglucose Positron Emission Tomography scan).

The lvPPA diagnosis was determined by clinical and medical imaging data according to the criteria by Gorno-Tempini et al. [11]. The patient had to present impaired single-word retrieval in spontaneous speech and naming, and an impaired repetition of sentences and phrases. The clinical diagnosis was reached when three of the following features were related: phonologic errors in spontaneous speech and naming, spared single-word comprehension and object knowledge, spared motor speech, absence of frank agrammatism. The GréMots tests associated with the Detection Test of Language impairments in Adults (DTLA) were used to objectify these clinical characteristics [47,48]. Imaging diagnosis was based on the detection of predominant left posterior perisylvian or parietal atrophy on MRI or predominant left posterior perisylvian or parietal hypoperfusion or hypometabolism on PET-SCANs [11,18]. In our study, the correlation of clinical and imaging diagnosis criteria validated lvPPA diagnosis. AD, considered as amnestic AD, was diagnosed by associated clinical, neuroimaging and CSF biomarkers analyses according to DSM-5TM criteria [46]. Clinical assessment corresponds to linguistic and neuropsychological tests. Imaging analyses were performed with MRI and PET-SCANs. Biomarker measures were obtained on a CSF sample by lumbar puncture.

Initially, 22 patients had met the inclusion criteria. However, 6 patients were excluded from the study because they refused to undergo the lumbar puncture. Therefore, the studied sample was composed of 8 patients with AD, and 8 patients with lvPPA, including 4 −lvPPA patients and 4 +lvPPA patients. The demographic and clinical descriptive data of the two groups and two sub-groups is summarized in Table 1. All patients were right-handed. Each patient from the AD group was matched with one patient from the lvPPA group, according to their age, educational level and cognitive level, as measured by the Minimal Mental State Examination (MMSE) score. All patients and relative caregivers only related cognitive difficulties. No behavioral or motor complaints were related.

### 2.3. Protocol Procedure

At their first consultation, patients orally received the necessary information for understanding the study and an informative note resuming these explanations. Then, patients signed the no-objection form at inclusion and investigators verified inclusion criteria. At the same time, paramedical examination was required for the patient to continue the study. An imaging exploration by MRI and/or PET-SCAN was performed. Patients had to undergo lumbar puncture to obtain CSF biomarker concentrations. If patients refused these additional examinations, they were excluded from the study. Then, anamnestic and descriptive data, resumed in Table 1, were collected. Anamnestic data include psychometric tests results: DTLA screening language impairment, MMSE precising cognition level, Instrumental Activities of Daily Leaving (IADL) scale as an autonomy score from 0 to 4, the *Batterie Rapide d’Efficience Frontale* (BREF, “rapid evaluation of frontal efficiency”), screening executive functions, and categorical fluency tested on animals to evaluate lexical access and verbal working memory. To score language impairment, the practitioner administered the DTLA [48]. Investigators added a sentence repetition task called “Sentence Span Test” (SST), the protocol for which is described on Appendix A [26]. The language assessment was audio-recorded. The assessment was held in a quiet room. The recorder was placed 10 cm in front of the patient and the microphone was oriented towards the patient’s mouth [44].

### 2.4. Material

The recording was made on an Apple iPad, recording stereo sound with two included microphones with the parameter “Audio Quality without loss”. The SST is a memory-intensive spoken repetition task including 14 sentences. The sentence stimuli had increasing span length content of words, ranging from a span of 3 content words (e.g., *Un étudiant fait ses devoirs*; “A student does his homework”.) up to 9 content words (e.g., *Les chercheurs en archéologie ont découvert une grande tombe romaine dont des squelettes et des outils tranchants.* “Researchers in archaeology have discovered a large Roman tomb including skeletons and sharp tools”.). There were two sentences per span length. Within a span task design, the SST measure targets verbal working-memory capacity during sentence repetition, which is an early symptom in lvPPA and AD patients. This facilitates an early diagnosis for AD and lvPPA patients [44]. The complete sentences included in the SST are reported in Table A1 in Appendix A.

Lumbar punctures allowed the extraction of CSF and measurement of AD relative biomarkers. Measures of β 42-Amyloïd peptide, total tau and phospho-tau protein concentrations were collected [49,50]. The standard for each protein corresponds to 700–1800 pg/mL, 130–6001 pg/mL and 20–60 ng/mL, respectively.

### 2.5. Acoustic Analyses

Acoustic analysis was carried out on the 14 repeated sentences in the SST, detailed in Table A1 in Appendix A. Sentences were recorded as .wav files. The recordings were cut into 28 extracts corresponding to the patient’s repeated sentence with or without the sentence’s model given by the investigator. This was achieved with Audacity 3.1.3 software [51]. Acoustic analyses were implemented with PRAAT software 6.2.06 [52]. Extracted acoustic and vocal parameters were chosen because of their significant impact on AD diagnosis from controls, as demonstrated in previous studies described in the literature [53,54]. An overview and explanation of extracted acoustic features are presented in Table 2. With PRAAT software, temporal and acoustic measures were implemented. Acoustic parameters correspond to a periodicity range from 75 to 600 Hz with the autocorrelation method.

### 2.6. Statistical Analyses

Descriptive statistics were used to present demographic and clinical characteristics. Qualitative variables (gender, educational level, PET-SCAN and MRI results) were presented using percentages, and quantitative variables (psychometric scores and CSF biomarker concentrations) were described using mean, standard deviation (SD) and range. The group differences were tested with non-parametric statistical tests. The Wilcoxon–Mann–Whitney test was used for quantitative descriptive data and the χ^2^ test was used for qualitative descriptive data. The diagnostic groups showed no differences between their descriptive variables.

Concerning the sentence repetition task, descriptive analyses were first realized on psychometric data. The ratios of semantic and phonological errors and the number of content words were calculated for each sentence of each patient. Then, the mean result for each group studied was calculated. The performance score was also calculated. This corresponds to the ratio of repeated sentences without mistake or omission on content words which are highlighted in Table A1 in Appendix A. The mean performance ratio was calculated for each group: AD group, lvPPA and its sub-groups including −lvPPA and +lvPPA. Then, statistical analyses were carried out on acoustic, extracted data. This corresponds to the average results of the 14 repeated sentences for each previously extracted acoustic and temporal parameter studied in this protocol. The acoustic and temporal parameter differences were analyzed with the Wilcoxon–Mann–Whitney test. Then, variation rate was calculated on significant parameters with mean parameter values for each group.

## 3. Results

### 3.1. Demographic and Clinical Information

#### 3.1.1. Demographic and Psychometric Information

The Characteristics and clinical information for each group are reported in Table 1. No significant differences in demographic and clinical information were found across the two groups. The AD population was older than the lvPPA population and the two groups comprised more women than men, corresponding to the pathologies’ demography described in the literature [50]. They had an equivalent cognitive level as shown by the MMSE score (*p* = 0.709). As expected, the AD group had a higher language level than the lvPPA group, even though the difference in DTLA score is not significant (*p* = 0.127) [4]. The categorical fluency task shows better scores for the lvPPA population and, principally in the −lvPPA subgroup. This relates to greater impairment in the AD population for lexical access and working memory. The BREF score shows a more important alteration of executive capacities in the AD than the lvPPA group and, even more, the −lvPPA sub-group. The IADL scale shows more altered autonomy in the AD than the lvPPA group. This corresponds to the definition of AD as a global cognitive impairment pathology, contrary to lvPPA, where symptoms are specifically restricted to language impairment at onset time. Nonetheless, no differences are significant. This confirms the difficulty of significantly differentiating these two pathologies at an early stage of neurodegeneration.

#### 3.1.2. Neuroimaging and CSF Information

100% of AD patients accepted taking part in an MRI and only 37.5 of this group accepted a PET-SCAN. In the lvPPA group, 87.5% of patients accepted taking part in an MRI and 50% accepted the PET-SCAN. This includes 100% and 50% of −lvPPA patients with MRI and PET-SCAN results, respectively, and 75% and 50% of +lvPPA patients with MRI and PET-SCAN results, respectively.

In the AD population, of those who accepted the MRI imaging, the collected data revealed bilateral hippocampal atrophy from stage 1 to 3 on 6 patients’ brains (75%), including two associated with cortico-sub-cortical atrophy (25%). Three patients (37.5%) also presented cortico-sub-cortical atrophy and two patients (25%) had a parietal atrophy. On AD population’s PET-SCANs, parietal (75%) and temporal (25%) hypometabolism was observed. These PET-SCAN results correlate with parietal and cortico-sub-cortical atrophy on MRI observations.

In the lvPPA population with MRI results, three patients (43%) presented hippocampal atrophy from grade 1 to 4, two patients (29%) had a parietal atrophy and one patient (14%) presented cortico-sub-cortical atrophy. In the lvPPA PET-SCAN results, 10% of the population presented standard geriatric results and 90% of the population presented a temporal and/or parietal hypometabolism.

In the −lvPPA subtype, MRI results revealed one standard geriatric profile (25%), one hippocampal atrophy at stage 1 (25%), one parietal atrophy (25%) and one cortico-sub-cortical atrophy (25%). PET-SCAN results revealed standard metabolism on 25% of the population and temporo-parietal hypometabolism in 50% of the population. Temporo-parietal hypometabolism was associated with hippocampal atrophy in the −lvPPA population. In the +lvPPA subtype, two hippocampal atrophies at stage 1 and 4 (75%) and one parietal atrophy (25%) were observed. PET-SCAN results revealed parietal hypometabolism and temporo-parietal hypometabolism for 50% of the population in each case.

These observations correspond to the neuro-imaging descriptions described in the literature for lvPPA and AD [10,11,27].

CSF biomarker results presented in Table 3 reveal significant differences on the β 42-amyloid biomarker for the comparison of −lvPPA and AD as well as the comparison of the two subtypes −lvPPA and +lvPPA. The β 42-amyloid biomarker is not significant for +lvPPA and AD or for the diagnostic groups AD and lvPPA. The tau biomarkers show no significant differences. Moreover, the opposition to LP by 27% of the total initial population reflects the difficulty of convincing patients to undergo this invasive examination in the context of neurodegenerative diagnosis searches, as described in literature [35,36].

### 3.2. The SST Record Associated with Temporal and Prosodic Markers, an Efficient Task for Diagnosis

Linguistic scores are reported in Table 4. The lowest linguistic scores were observed in the lvPPA group. Concerning the mean number of repeated content words, the lvPPA group presented fewer content words. Semantic errors were mostly noticed in the AD group, in contrast to phonological errors, which more present in lvPPA repetitions. The same ratios were obtained concerning CSF biomarkers: in the lvPPA group, −lvPPA repetitions contained fewer content words and +lvPPA repetitions contained more semantic errors. Statistics reveal that phonological errors had the same proportion for the two lvPPA sub-groups.

Analyzing the performance scores of each group on the 14 sentence repetitions of the SST, a degree of variability in group performance for each sentence was observed. Errors began to appear in sentence 10 for AD and in sentence 5 for lvPPA. Concerning the lvPPA subtypes, errors were observed for the two sub-groups from sentence 4. Nonetheless, −lvPPA presented the lowest mean score with only 25% of the population succeeding in repetition of the 3 last sentences. Considering the −lvPPA and the +lvPPA subgroups, a difference of performance was observed from the sixth sentence onwards. A significant distinction regarding these two subtypes could be observed from a syntactic level all along the test when considering the lvPPA and AD diagnostic groups.

### 3.3. Acoustic Markers of Interest for AD and lvPPA Differential Diagnosis

For the temporal markers chosen for this study, Mann–Whitney statistics revealed a significant impact of temporal parameters. Table 5 presents mean values on temporal parameters for each group in our study. A significant difference is observed on patients’ reaction time to differentiate lvPPA and AD (*p* = 0.016). More precisely, lvPPA showed a mean reaction time increase of 76.79% compared to AD. The difference between the mean speech rates was not significant, considering pulses (*p* = 0.186), words (*p* = 0.186), syllables (*p* = 0.215) and phonemes (*p* = 0.159) per second.

Statistical analyses of temporal markers also revealed differences in pauses during repetition. AD patients presented a higher pauses rate than lvPPA, considering the number of total pauses, silent and non-silent pauses per second. Indeed, a significant difference was observed for the mean total pause rate (*p* = 0.033), the mean silent pause rate (*p* = 0.033) and the mean non-silent pauses rate (*p* = 0.063). AD patients showed a mean silent pause rate 64.15% higher than lvPPA patients and a mean non-silent pause rate 24.38% higher than the lvPPA group. The highest difference was found for total pause rate, with the AD patients’ mean rate 185.09% higher than for the lvPPA group. Other pause parameters, such as percentage cumulated duration or mean and standard deviation of pause duration, did not reveal any significant differences. Analyses revealed significant differences between the lvPPA and AD groups on mean occlusive consonant duration distance from the model (*p* = 0.042) and on mean fricative consonant duration distance from the model (*p* = 0.016). The lvPPA mean occlusive consonant distance from the model was 26.86% higher than for AD, and the lvPPA mean fricative consonant gap was 30.84% higher than for AD. The difference between the raw mean measurement of occlusive consonants duration tends to significance (*p* = 0.051) and the difference between the raw mean measurement of fricative consonants is significant (*p* = 0.041). Mean duration of consonants is longer for lvPPA than AD, with a 37.72% increase for occlusive consonants duration and 36.75% for fricatives. Vowel phonation time showed no significant differences with raw measurements (*p* = 0.356) or duration distance from the model (*p* = 0.437).

From the acoustic markers studied here, Mann–Whitney statistics revealed significant differences on prosodic parameters. Table 6 presents mean values on prosodic parameters for each group in our study. Firstly, analyses revealed significant differences in periodicity variations. Mean maximum F0 (*p* = 0.026) and F0 range trend to significance on the mean F0 range (*p* = 0.051). AD patients presented a greater F0 range, with a difference of 20.62% and a higher maximum F0 increase of 17.43% in AD patients compared to lvPPA patients.

To resume the previous comparisons, the most relevant temporal parameters to distinguish lvPPA and AD patients at the onset time of the diseases are the reaction time, the pauses ratios, including non-silent and silent pauses ratios, and the occlusive and fricative consonant mean duration distances from the model. The most relevant acoustic parameters are the F0 variation and principally the mean maximum F0.

### 3.4. Acoustic and Temporal Characteristics according to Biomarkers

#### 3.4.1. Acoustic and Temporal Differences in lvPPA Group Sub-Types According to CSF Biomarkers Variations

Mann–Whitney statistics demonstrated that none of the temporal parameters significantly differentiated −lvPPA and +lvPPA subgroups. Nonetheless, statistical analyses revealed that relevant prosodic markers could distinguish the two subgroups. The two subgroups were significantly distinguished by mean minimum F0 (*p* = 0.030). The +lvPPA subgroup presented a higher minimum F0, 23.41% higher than −lvPPA. None of the other parameters were highlighted as significant with Mann–Whitney statistics.

#### 3.4.2. Comparison of Acoustic and Temporal Features, Diagnostic Groups, and Their Biomarker Profiles

According to statistical analyses, the +lvPPA subgroup was distinctive from AD in terms of temporal markers. Only one parameter shows significant differences with the AD group: the patients’ mean reaction time (*p* = 0.017). The +lvPPA subgroup presents a mean reaction time 136.12% higher than the AD group. A difference trending to significance was observed on fricative consonant duration (*p* = 0.053) and its distance from model duration (*p* = 0.053). The +lvPPA subgroup has a fricative consonant mean duration and distance from the model duration 26.24% and 24.95% higher than for the AD group, respectively. None of the acoustic parameters shows a significant distinction between +lvPPA and AD.

Concerning the −lvPPA group, statistical tests revealed a significant difference of patients’ reaction time compared to the AD group (*p* = 0.010). Reaction time is significantly higher, by 96.32%, in the −lvPPA group. A significant distinction is also shown for fricative and occlusive (*p* = 0.037, *p* = 0.037) consonant duration and their distance from models (*p* = 0.025, *p* = 0.037). Fricative and occlusive consonants in the lvPPA group have a higher duration, 53.33% and 42.49% higher than for the AD group, respectively. Pause ratio also shows relevant differences between −lvPPA and AD, principally for total pause ratio (*p* = 0.037) and silent pause ratio (*p* = 0.025). Both AD ratios were higher than the −lvPPA ones, with respective differences of 186.54% and 66.87%. Finally, maximum F0 significatively differentiates −lvPPA from AD (*p* = 0.053), with maximum F0 that was 13.25% higher.

**Figure 1:**
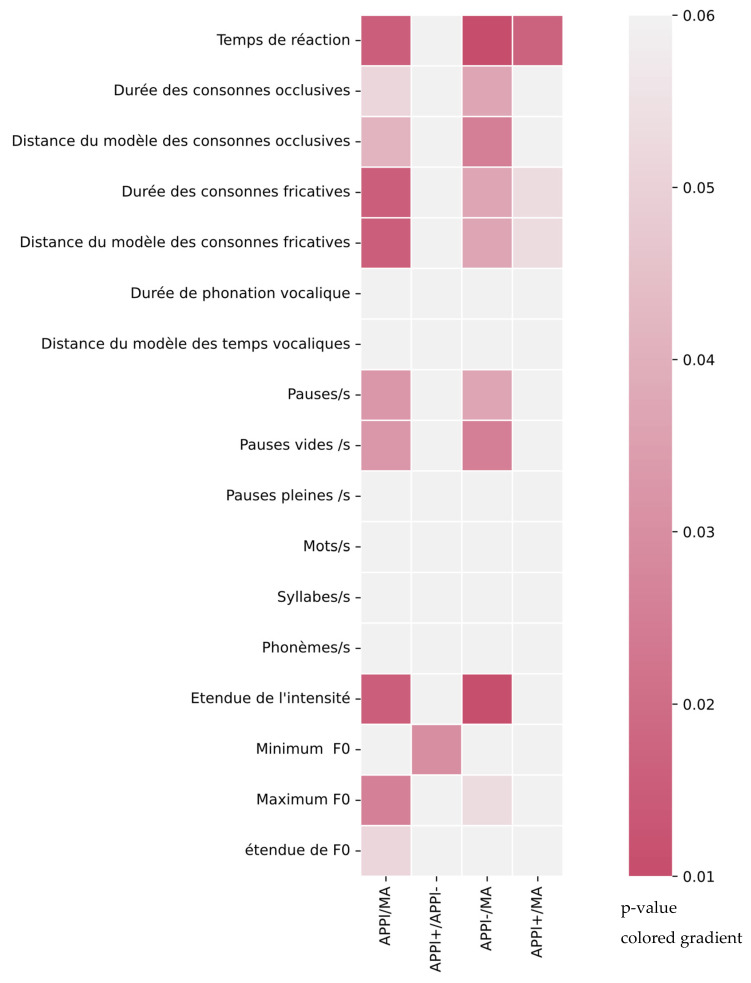
Distribution of significant values on acoustic parameters represented by colored gradient. Significance degree of each parameter for lvPPA and AD, +lvPPA and −lvPPA, +lvPPA and AD, −lvPPA and AD comparisons.

To resume previous comparisons, +lvPPA can only be differentiated from AD by patients’ mean reaction time, fricative consonant mean duration, and its distance from model mean duration. In parallel, −lvPPA significatively differs from AD for maximum F0. Temporal features vary for total and silent pause ratios, patients’ mean reaction time and fricative and consonant durations as well as their distance from models. A summary of the distribution of significant values on acoustic parameters is represented in Figure 1.

The figure represents a colored gradient of *p*-values for the significance of each acoustic parameter and each comparison according to statistical tests. It relates one acoustic parameter, named on the y-axis, with a comparison of two groups, named on the x-axis. According to the Mann–Whitney test, the difference between the two groups is considered as significant when the *p*-value is less than 0.05.

## 4. Discussion

In the present study, we investigated the usefulness of acoustic parameters collected on a memory-intensive sentence repetition protocol with the SST to differentiate patients with lvPPA from patients with AD at onset time. We also compared their CSF biomarker profile to their acoustic profile.

The interest of acoustic parameters for accurate diagnosis between the lvPPA and AD groups was demonstrated in this study. Indeed, the temporal analyses, performed on the SST outcomes, showed significant features for discriminating the two groups. Differences were found on the mean fricative and occlusive consonant duration, with a greater duration in the lvPPA group compared to the AD group. Previous studies showed no significant variations on AD occlusive consonant voice onset time compared with healthy controls and, on the contrary, an elongation of vowel phonation duration was associated with cognitive decline [55,56]. Our results reveal an increase in consonant voice onset time in lvPPA speech, in contrast to AD. As the two groups have equivalent mean vowel mean durations, a pathological elongation of vowels in the AD group appears here as equivalent to lvPPA at onset time.

Moreover, model temporal deviation was also observed in previous studies on AD and in lvPPA comparisons to other PPA subtypes [44,59]. Our study also exposed a temporal deviation from models in the two groups, with a significant increase in deviation from model production in lvPPA occlusive and fricative consonant duration. Phonological loop alteration, a specific symptom of the lvPPA profile, would explain these specific alterations, differentiating the lvPPA from AD group by the slowing down of consonant production [60]. Consequently, the voice-onset time of consonants increases due to a phonological loop deficit [56]. Phonological loop alteration would also explain the patient reaction time augmentation observed here through an extended vocal initiation time on lvPPA repetitions.

Our results also differentiate lvPPA from AD on the base of pause rates. Pauses permit the differentiation of AD and lvPPA from healthy controls, with an increase in pause frequency [57,61]. Our study, comparing these two groups, showed an impact of total silent and non-silent pause rates on their discrimination. Differing from a previous study, the AD profile here showed the higher pause rate [62]. The greatest difference for AD was observed on silent pause rate. Indeed, AD has been described in the literature as featuring numerous silent pauses. On the contrary, lvPPA has been described to feature non-silent pauses such as autocorrections, repetitions or voiced hesitations. Pauses were previously interpreted in the literature as a compensatory mechanism for lexico-semantic and working memory decline at an early stage of AD [57]. Thus, these temporal alterations allow for a clear differentiation between lvPPA and AD at onset time.

Prosody analyses also show a high discriminant value in our study. F0 appears here as an important acoustic marker in AD and lvPPA detection, with significant differences for F0 range and maximum F0. In our study, the AD group presented a larger F0 interval and a higher F0 on average. Fundamental frequency and its evolution have already been considered as a marker of interest for differentiating patients with AD and lvPPA from healthy controls [4,55,58]. This parameter is related to emotional prosody. F0 alteration in AD has been correlated to the decrease in F0 variations whereas, here, the AD profile presents the greatest F0 range and the highest maximum F0 [58,63]. Thus, F0 comparisons show a higher alteration in the lvPPA group compared to the AD group. In addition, the intensity amplitude range stood out as a key feature for clustering the two groups. It appears that, in our study, the AD profile had a larger intensity amplitude than the lvPPA profile. However, the literature demonstrating AD acoustic diagnostic markers include a low intensity range, inducing monotonous speech compared to healthy controls [64]. Prosody in lvPPA has also shown to exhibit alterations in a vocal sentence reading task [65]. Therefore, our results suggest a more important prosodic alteration, illustrated by low intensity and fundamental frequency, in the lvPPA group compared to the AD group in an intensive repetition task at onset time.

Considering CSF biomarkers, two lvPPA subgroups are formed: +lvPPA and −lvPPA. Here, β-Amyloid markers present a significative difference, suggesting two clinically different lvPPA populations. Moreover, CSF biomarker groups are in accordance with acoustic specificities. Each one revealed a specific acoustic profile which would permit clustering each population early according to their CSF biomarkers and clinical profiles thanks to acoustic measures.

The +lvPPA subgroup corresponds to an Alzheimer-disease-like CSF profile with positive β-Amyloid markers [25,33,34]. According to our results, +lvPPA is only different in temporal parameters: reaction time, fricative and occlusive consonant phonation time and their distance from model. These temporal parameters were higher than in AD on average. Therefore, our results suggest that +lvPPA has an acoustic profile like AD, except on temporal features. In the literature, lvPPA has been described to have specific alteration regarding the phonological loop and lexical processing [18,60]. These cognitive functions are involved in the affected temporal parameters such as consonant production duration or reaction time [66]. Atypical AD with a primary language alteration on lexical access and sentence repetition has already been described in the literature [67]. Thus, our results raise the hypothesis that the +lvPPA acoustic profile correlates with an atypical form of AD, with first symptoms focused on language, and specifically on phonological short-term memory and lexical access alteration [66]. This is supported by the imaging description of the +lvPPA and AD groups in our study cohort. Effectively, AD and +lvPPA have correlated neuro-imaging descriptions: the same proportion of hippocampal atrophy was observed in the +lvPPA and AD groups. This hypothesis must be verified by future studies with a greater sample of the lvPPA and AD populations including corresponding CSF biomarkers data. These results also emphasize the interest of studies on +lvPPA progression over time in order to determine an eventual convergence with specific AD with predominant language impairment.

The −lvPPA group differs in terms of similar acoustic parameters from AD as the lvPPA diagnostic group described before. Compared to AD, the −lvPPA group presents a lower maximum fundamental frequency, intensity range, and total pause and silent pause ratios. Consonant phonation and distance from the model as well as reaction time were also more altered in the −lvPPA compared to AD. These acoustic alterations were greater than in the lvPPA diagnostic group. Furthermore, the literature describes similar acoustic alterations in FTLD. FTLD silent pause rate is significantly higher compared to healthy controls [68]. An evolution of FTLD pause rate is described on the same continuum as the lvPPA pause rate increase [69]. FTLD prosody alteration is also similar: the reduction of the fundamental frequency range has been described [70]. In addition, the −lvPPA group presented the highest alteration of prosodic parameters, and prosodic alteration is correlated to frontal and parietal lobes, which are neuropathological observations associated with FTLD [65]. Consequently, the −lvPPA acoustic profile reported here correlates with FTLD acoustic characteristics presented in the literature. Nonetheless, the −lvPPA acoustic characteristics must be confirmed with a larger sample of the population. Moreover, neuro-imaging characteristics of the −lvPPA group studied do not completely correspond to FTLD anatomopathology. Thus, the hypothesis correlating −lvPPA with an underlying FTLD can be verified by analyzing acoustic markers of −lvPPA with an underlying FTLD evolution over time.

Therefore, acoustic analyses comparing lvPPA and AD associated with CSF biomarker profiles allow us to reconsider the PPA classification consensus. The literature has already questioned the actual classification, analyzing PPA types of properties with automatic clustering algorithms [3,11]. Our results correlated lvPPA biomarker profiles with acoustic profiles, dividing them into two acoustic patterns. Future studies may confirm the hypothetic correlation of each acoustic profile to a specific underlying pathology.

These acoustic profiles are also coherent with MRI and PET-SCAN anatomic markers associated with each lvPPA CSF biomarker type and underlying pathology. The +lvPPA anatomopathological form presents gray matter atrophy in the temporal gyrus and parietal region associated with a parietal hypometabolism which is consistent with AD. On the contrary, −lvPPA is characterized in the literature by alterations located in the left anterior temporal region and the left inferior frontal-insula region, similarly to atrophied regions in FTLD [25,31,32]. Nonetheless, the −lvPPA population studied only presented a temporal hypometabolism on PET-SCANs which can be associated with an underlying FTLD. Other similarities cited in the literature were not observed in this population. Nonetheless, prosodic acoustic alterations observed in the −lvPPA group are associated with the left lateral temporal lobe [65]. PET-SCAN alterations, targeting phonological loop deficits, corroborate with temporal features alterations observed in −lvPPA in the sentence repetition task [25]. Thus, the two different acoustic profiles revealed by acoustic analyses on lvPPA subgroups correspond to the two MRI neuro-anatomic profiles described in the literature [31,32]. Our study thereby supports the same hypothesis of two different lvPPA subtypes differing in language, neuroanatomic alterations, CSF biomarkers and early acoustic markers. The lack of neuroanatomic deficits described in the literature in the population studied may be due to the examination being completed at an early stage of the pathology. It can be verified by an analysis of the clinical evolution of the lvPPA subgroups associated to their anatomopathological progression over time.

Moreover, this study offers new possibilities for the differential diagnosis of neurodegenerative pathologies at onset time. Until now, no significant marker has been described in the literature to specifically distinguish these two groups. Paramedical tests such as LP are not sufficiently accurate and, even more importantly, are not sufficiently accepted by patients [20,29]. Therefore, the use of discriminant acoustic features as diagnostic tools would permit a precise differential diagnosis between lvPPA and AD. If our results are verified with a larger population, we would consider acoustic markers as effective prognostic factors for estimating a CSF biomarker profile for each patient with a suspicion of AD or PPA. Nonetheless, a larger sample of the population is necessary to confirm this preliminary study and to verify these results.

Despite this limitation, this study offers great opportunities considering the diagnosis precision in association with patient well-being. On our research continuum, the elaboration of an automated classification thanks to automatic acoustic data extraction and artificial intelligence algorithms would permit the creation of a non-invasive, early, accessible and precise diagnosis tool [45]. The precision would be ensured by the complete automatization of the system and the SST protocol by the implication of a phonological loop on its assessment [20].

Firstly, the SST, an intensive sentence repetition task, appeared as relevant for discriminating lvPPA from AD with acoustic analyses. This study confirmed the interest of an acoustic analysis of oral sentence repetition, with an increasing difficulty, as a discriminant feature for lvPPA and AD patients at onset time. The literature has already shown differences between lvPPA and AD on the SST, even if the results are not sufficiently clear-cut to obtain a differential diagnosis [20]. As observed in our results, lvPPA scores were lower than AD ones considering psycholinguistic measurements. Thus, using acoustic analyses on a task targeting lvPPA deficits seems to permit a significant differentiation between the two groups in terms of acoustic analyses. Indeed, previous studies have already shown an improvement over pencil-paper assessment by using acoustic analyses to detect significant differences not achievable by natural hearing or global and duration scores [32].

Secondly, acoustic data analyses would permit a rapid screening prognosis. This acoustic prognosis would also specify a first patient’s diagnosis or serve as an alternative tool when opposition to invasive tests such as LP is expressed by patients. Indeed, previous studies have reported a lack of precision of AD diagnosis and the numerous side-effects impacting the patient’s well-being and motivation to take LP examination [27,28]. In addition, LP is marked by high cost and an inequity of access between patients’ places of residence, as pointed out in the literature [27]. Therefore, developing associated noninvasive diagnostic instruments to complement the actual diagnosis tools would facilitate diagnosis accessibility and patients’ rapid and motivated inclusion in an adapted care project. In accordance with these previous reasons, continuing the research on a diagnostic acoustic assessment will facilitate AD and lvPPA diagnostic access.

Integrating acoustic markers in lvPPA and AD diagnosis would offer opportunities for an early and adapted care which would reduce disease evolution and encourage patients’ abilities to be maintained over time. Indeed, acoustic markers would complement psychometric evaluations such as DTLA to precisely identify neuropathologies [39].

## 5. Conclusions

To conclude, acoustic markers enable the early differential diagnosis of patients with lvPPA and AD. Temporal and prosodic parameters revealed significant differences in an assessment targeting the lvPPA-specific phonological loop deficit. Acoustic markers in the SST compared to the CSF biomarker profiles also highlighted new possibilities for making PPA classification more precise. Acoustic marker analyses presented here conform with the hypothesis, stated in the literature, regarding two potential lvPPA subtypes and the possibility of classification according to MRI observations, CSF biomarkers and acoustic markers. In this regard, the lvPPA classification including CSF biomarkers, associated with our preliminary acoustic results, offers the possibility of an analysis by acoustic markers used as an accuracy test to complement actual assessment. It would serve to classify both lvPPA subtypes and AD patients. Acoustic markers would also serve as an alternative prognosis tool in cases of opposition to invasive examinations such as lumbar puncture. Finally, these new discoveries improve the comprehension of PPA syndrome and its links with other neurodegenerative pathologies.

## Figures and Tables

**Table 1:** Descriptive data of the population.

	AD	lvPPA	−lvPPA	+lvPPA	*p*-Value
N	8	8	4	4	
Female (%) *	62.5	62.5	50	75	1
Age range (y)	67–86	52–77	52–77	61–77	
mean age **	74.875	69.5	67.75	71.25	0.26
SD age	6.17	8.97	11.35	7.13	
Laterality (Right %) *	100	100	100	100	1
Primary Education level (%) *	25	25	0	50	1
Secondary Education level (%) *	62.5	62.5	75	50	1
Higher Education level (%) *	12.5	12.5	25	0	1
range MMSE	21–27	20–28	20–28	20–28	
mean MMSE **	23	23.75	23	24.5	0.71
SD MMSE	2.32	3.41	3.82	3.31	
Range DTLA score	61–98	57–94	66–94	57–88	
mean DTLA score **	86.75	78.00	78.25	77.75	0.13
SD DTLA score	12.25	12.05	11.67	14.24	
range BREF score	11–18	9–18	14–18	9–17	
mean BREF score **	14.25	14.62	16.25	13.00	0.87
SD BREF score	3.23	2.95	1.48	3.16	
Range categorical fluency	4–29	18–40	24–40	18–29	
mean categorical fluency **	19.00	29.38	34.75	24.00	0.07
SD categorical fluency	9.03	7.61	6.53	3.94	
mean IADL score **	1.63	0.38	0.25	0.50	0.09
SD IADL score	1.49	0.70	0.43	0.87	

* χ²; ** Kruskal–Wallis, *p*-values refer to the overall comparisons between AD and lvPPA.

**Table 2:** Overview and explanation of extracted acoustic features.

Features	Explanations	Category
Vocal reaction time	Latency time before initiating sentence repetition(s). [43]	Temporal
Vowel phonation time	Mean vowel phonation duration (s). [55]	Temporal
Consonant phonation time	Distinctive mean occlusive and fricative consonant voice onset time and sounding time (s). [56]	Temporal
Phonation time deviation from model	Phonation time distance compared to model’s phonation time (calculated on vowels, occlusive and fricative consonants). [44]	Temporal
Pause ratio	Number of total pauses per second. [53]	Temporal
Non-silent pause ratio	Number of non-silent pauses, considering hesitations, autocorrections and repetitions. [53]	Temporal
Silent pause ratio	Number of silent pauses, considering voiceless segments longer than 30 ms. [53,57]	Temporal
Speech rate	Number of pulses/words/syllables/phonemes per second. [53]	Prosodic
Intensity range	Distance between maximum and minimum intensity amplitude during sentence repetition (dB).	Prosodic
Fundamental frequency (F0) maximum range	Distance between maximum and minimum fundamental frequency during sentence repetition (Hz). [58]	Prosodic
Minimum F0	Lowest fundamental frequency during sentence repetition (Hz). [58]	Prosodic
Maximum F0	Highest fundamental frequency during sentence repetition (Hz). [58]	Prosodic

**Table 3:** CSF biomarkers concentration, extracted by lumbar puncture.

	β 42-Amyloid (±SD)	Total Tau (±SD)	Phospho-Tau (±SD)
**AD**	636.5 (±173.64)	874.625 (±234.59)	166.625 (±38.28)
**lvPPA**	901.375 (±687.29)	495.5 (±447.25)	90.375 (±128.46)
**−lvPPA**	1371.75 (±747.24)	494.25 (±571.69)	79.75 (±170.58)
**+lvPPA**	431 (±97.131)	496.75 (±133.36)	101 (±28.40)
***p*** **-value** **AD/lvPPA**	0.38	0.38	0.72
***p*** **-value** **AD/+lvPPA**	0.36	0.21	0.68
***p*** **-value** **AD/−lvPPA**	0.008 *	0.93	0.93
***p*** **-value** **+lvPPA/−lvPPA**	0.05 *	0.68	1

* Kruskal–Wallis, *p*-values refer to the overall comparisons between the groups cited in the 1st column.

**Table 4:** Mean linguistic score on the 14 SST sentences.

Diagnoses	N	Mean Content Words (±SD)	Mean Semantic Errors (±SD)	Mean Phonological Errors (±SD)	Mean Performance Score Ratio (±SD)
**AD**	8	4.169 (±1.432)	0.161 (±0.216)	0.018 (±0.042)	0.857 (±0.240)
**lvPPA**	8	4.034 (±0.798)	0.299 (±0.325)	0.055 (±0.076)	0.786 (±0.179)
**+lvPPA**	4	4.452 (±0.855)	0.457 (±0.501)	0.054 (±0.099)	0.857 (±0.857)
**−lvPPA**	4	3.643 (±0.855)	0.161 (±0.235)	0.054 (±0.099)	0.696 (±0.696)

Group and sub-group characteristics correspond to the ones described in Table 1.

**Table 5:** Mean temporal parameters for the AD and lvPPA population.

Temporal Parameters	Significance (*p*-Value)	Diagnoses	Mean (±SD)
Vocal reaction time (s)	AD/lvPPA (0.016) **	AD	0.779 (±0.180)
	AD/−lvPPA (0.010) **	lvPPA	1.379 (±0.727)
	AD/+lvPPA (0.017) **	+lvPPA	1.842 (±0.751)
	−lvPPA/+lvPPA (0.096)	−lvPPA	1.916 (±0.253)
Occlusive consonants duration (s)	AD/lvPPA (0.051) *	AD	0.049 (±0.016)
	AD/−lvPPA (0.037) **	lvPPA	0.067 (±0.017)
	AD/+lvPPA (0.097)	+lvPPA	0.101 (±0.012)
	−lvPPA/+lvPPA (0.222)	−lvPPA	0.113 (±0.016)
Occlusive consonant distance from model	AD/lvPPA (0.042) **	AD	1.083 (±0.397)
	AD/−lvPPA (0.025) **	lvPPA	1.481 (±0.389)
	AD/+lvPPA (0.156)	+lvPPA	1.288 (±0.304)
	−lvPPA/+lvPPA (0.222)	−lvPPA	1.674 (±0.368)
Fricative consonant duration (s)	AD/lvPPA (0.016) **	AD	0.079 (±0.022)
	AD/−lvPPA (0.037) **	lvPPA	0.107 (±0.015)
	AD/+lvPPA (0.097)	+lvPPA	0.101 (±0.012)
	−lvPPA/+lvPPA (0.053) *	−lvPPA	0.114 (±0.015)
Fricative consonant distance from model	AD/lvPPA (0.016) **	AD	0.978 (±0.261)
	AD/−lvPPA (0.037) **	lvPPA	1.274 (±0.155)
	AD/+lvPPA (0.235)	+lvPPA	1.223 (±0.138)
	−lvPPA/+lvPPA (0.053) *	−lvPPA	1.326 (±0.154)
Vowel phonation time (s)	AD/lvPPA (0.357)	AD	0.153 (±0.020)
	AD/−lvPPA (0.222)	lvPPA	0.154 (±0.022)
	AD/+lvPPA (0.156)	+lvPPA	0.145 (±0.018)
	−lvPPA/+lvPPA (0.466)	−lvPPA	0.162 (±0.022)
Vowel phonation distance from model	AD/lvPPA (0.437)	AD	1.195 (±0.148)
	AD/−lvPPA (0.399)	lvPPA	1.171 (±0.153)
	AD/+lvPPA (0.333)	+lvPPA	1.133 (±0.156)
	−lvPPA/+lvPPA (0.466)	−lvPPA	1.208 (±0.139)
Pauses/s	AD/lvPPA (0.033) **	AD	2.175 (±0.565)
	AD/−lvPPA (0.037) **	lvPPA	0.763 (±0.387)
	AD/+lvPPA (0.443)	+lvPPA	0.7669 (±0.450)
	−lvPPA/+lvPPA (0.135)	−lvPPA	0.759 (±0.309)
Silent pauses/s	AD/lvPPA (0.033) **	AD	1.148 (±0.316)
	AD/−lvPPA (0.037) **	lvPPA	0.699 (±0.410)
	AD/+lvPPA (0.442)	+lvPPA	0.711 (±0.480)
	−lvPPA/+lvPPA (0.175)	−lvPPA	0.688 (±0.325)
Non-silent pauses/s	AD/lvPPA (0.054) *	AD	0.227 (±0.311)
	AD/−lvPPA (0.175)	lvPPA	0.058 (±0.028)
	AD/+lvPPA (0.333)	+lvPPA	0.056 (±0.031)
	−lvPPA/+lvPPA (0.074)	−lvPPA	0.059 (±0.024)

(*p*-value) ** Significance according to Mann–Whitney tests; (*p*-value) * a comparison tending to significance.

**Table 6:** Mean prosodic parameters for the AD and lvPPA population.

Prosodic Parameters	Significance (*p*-Value)	Diagnosis	Mean (±SD)
Words/s	AD/lvPPA (0.186)	AD	2.327 (±0.383)
	AD/−lvPPA (0.134)	lvPPA	1.928 (±0.604)
	AD/+lvPPA (0.333)	+lvPPA	2.179 (±0.253)
	−lvPPA/+lvPPA (0.399)	−lvPPA	1.677 (±0.735)
Syllables/s	AD/lvPPA (0.215)	AD	3.676 (±0.580)
	AD/−lvPPA (0.222)	lvPPA	3.002 (±0.990)
	AD/+lvPPA (0.333)	+lvPPA	3.379 (±0.439)
	−lvPPA/+lvPPA (0.336)	−lvPPA	2.625 (±1.219)
Phonemes/s	AD/lvPPA (0.159)	AD	7.536 (±1.216)
	AD/−lvPPA (0.175)	lvPPA	6.118 (±2.052)
	AD/+lvPPA (0.442)	+lvPPA	6.913 (±0.896)
	−lvPPA/+lvPPA (0.276)	−lvPPA	5.324 (±2.521)
Intensity range (dB)	AD/lvPPA (0.016) **	AD	35.920 (±5.860)
	AD/−lvPPA (0.011) **	lvPPA	27.036 (±5.249)
	AD/+lvPPA (0.097)	+lvPPA	545.932 (±54.823)
	−lvPPA/+lvPPA (0.135)	−lvPPA	24.094 (±4.168)
Minimum F0 (Hz)	AD/lvPPA (0.396)	AD	95.547 (±17.999)
	AD/−lvPPA (0.101)	lvPPA	87.088 (±15.750)
	AD/+lvPPA (0.030) **	+lvPPA	98.634 (±9.661)
	−lvPPA/+lvPPA (0.222)	−lvPPA	75.542 (±11.669)
Maximum F0 (Hz)	AD/lvPPA (0.026) **	AD	382.273 (±91.249)
	AD/−lvPPA (0.053) *	lvPPA	325.541 (±49.334)
	AD/+lvPPA (0.442)	+lvPPA	319.474 (±64.704)
	−lvPPA/+lvPPA (0.074)	−lvPPA	331.605 (±24.646)
F0 range (Hz)	AD/lvPPA (0.051) *	AD	271.015 (±91.946)
	AD/−lvPPA (0.101)	lvPPA	224.689 (±46.748)
	AD/+lvPPA (0.442)	+lvPPA	208.469 (±61.490)
	−lvPPA/+lvPPA (0.102)	−lvPPA	240.91 (±7.964)

(*p*-value) ** Significance according to Mann–Whitney tests; (*p*-value) * a comparison tending to significance.

## Data Availability

The data reported are part of an ongoing registration program. Deidentified participant data are not available for legal and ethical reasons. Anonymized data will be made available for research purposes, upon request and specifical approval of the database advisory board and ethical committee.

## Appendix A

### Protocol Procedure

Put the recorder on the table, 10 cm from the patient. The microphone has to be placed towards patient’s mouth. Record all the assessment.

Recite the following guideline to the patient:

«Je vais vous dire des phrases, répétez-les après moi.» (“I am going to tell you sentences, repeat after me”)

**Table A1:** The SST protocol and its English translation.

Item	Span (Total Words)	Stimulus
1	3 (5)	*La **fille mange** un **gâteau.*** “The girl eats a cake”
2	3 (5)	*Un **étudiant fait** ses **devoirs.*** “A student does his homework”.
3	4 (6)	***L’enseignant******part** et **ferme** la **porte.*** “The teacher leaves and closes the door”.
4	4 (7)	*Le **chat** a **chassé** une **PET-scanite souris.*** “The cat chased a little mouse”.
5	5 (8)	*Les **oiseaux** sont **nichés** sur un **grand arbre vert.*** “The birds are nestled on a tall green tree”.
6	5 (9)	*La **mère** du **garçon** a **acheté** des **amandes** au **marché***. “The boy’s mother bought almonds at the market”.
7	6 (11)	*Une **jeune fille a tenté** un **plongeon** pendant ses **vacances** en **juillet.*** “A young girl attempted a dive while on vacation in July”.
8	6 (12)	*Le **vieil homme** est en **retard** alors il **veut prendre** un **taxi**.* “The old man is late so he wants to take a taxi”
9	7 (13)	***L’évier****de la **cuisine** est **très bouché** mais le **réparateur utilise** une **pompe.*** “The kitchen sink is very clogged but the repairman uses a pump”.
10	7 (14)	Au **restaurant** la **femme** du **professeur** a **mangé** des **nouilles asiatiques** pour le **déjeuner.** “At the restaurant the teacher’s wife ate Asian noodles for lunch”.
11	8 (16)	*Le **lapin** s’est **échappé très vite** dans la **forêt** et le **chasseur** n’a pas **réussi** à **l’attraper.*** “The rabbit escaped very quickly into the hood and the huntman failed to catch him”.
12	8 (16)	*Les **touristes** ont **découvert** les **immenses sculptures** de **bronze** du **musée** grâce à la **visite guidée**.* “Tourists discovered the museum’s huge bronze sculptures through the guided tour”.
13	9 (17)	*Les **chercheurs** en **archéologie** ont **découvert** une **grande tombe romaine** dont des **squelettes** et des **outils tranchants**.* “Archaeological researchers have discovered a large Roman tomb including skeletons and sharp tools”.
14	9 (18)	*Les **océans deviendront** nos **ennemis** si **rien** n’est **fait** pour **freiner** le **réchauffement** dû aux **émissions** de **gaz**.* “The oceans will become our enemies if nothing is done to curb the warming caused by gas emissions”.
